# A screen printed carbon electrode modified with carbon nanotubes and gold nanoparticles as a sensitive electrochemical sensor for determination of thiamphenicol residue in milk†

**DOI:** 10.1039/c7ra07544h

**Published:** 2018-01-11

**Authors:** Aliyu Muhammad, Reza Hajian, Nor Azah Yusof, Nafiseh Shams, Jaafar Abdullah, Pei Meng Woi, Hamid Garmestani

**Affiliations:** Department of Chemistry, Faculty of Science, Universiti Putra Malaysia 43400 Serdang Selangor Malaysia azahy@upm.edu.my; School of Materials Science and Engineering, Georgia Institute of Technology Atlanta GA 30332 USA reza.hajian@mse.gatech.edu; Institute of Advanced Technology, Universiti Putra Malaysia 43400 Serdang Selangor Malaysi; Department of Chemistry, Faculty of Science, University of Malaya 50603 Kuala Lumpur Malaysia

## Abstract

Antibiotic residues in milk are of great concern for health regulatory agencies, milk consumers, and dairy farmers due to their destructive effects, ranging from allergic reactions, antibiotic resistance and the ability to interfere with the production of fermented products (*i.e.* cheese and yogurt). Therefore, a reliable, fast, and simple method needs to be developed to monitor antibiotic residues in milk samples before distribution to consumers. In this study, the first sensitive electrochemical sensor is presented for the determination of thiamphenicol (TAP), a broad-spectrum antibiotic in bovine milk. In the fabrication process, a screen printed electrode (SPE) was modified with gold nanoparticles (AuNPs) and carbon nanotubes (CNTs) using ethylenediamine (en) as a cross linker. Cyclic voltammetry studies showed an adsorptive control process for the electro-oxidation of TAP at −0.1 V on the modified electrode of SPE/CNT/en/AuNPs. Differential pulse voltammetry (DPV) was applied for the quantitative determination of TAP under optimized conditions (0.1 M citrate buffer, pH 6.0, accumulation potential −0.7 V, and accumulation time 150 s). A DPV study for TAP shows a wide linear calibration range of 0.1–30 μM with the detection limit of 0.003 μM. Furthermore, the developed sensor displays high sensitivity, reproducibility, repeatability, and good stability for the detection of TAP. The proposed sensor was successfully applied for the determination of spiked TAP in bovine milk with satisfactory results.

## Abstract 2

The first portable electrochemical sensor was constructed for monitoring of thiamphenicol residue in fresh milk based on electro-oxidation of thiamphenicol on the surface of CNTs and AuNPs.

## Graphical abstract

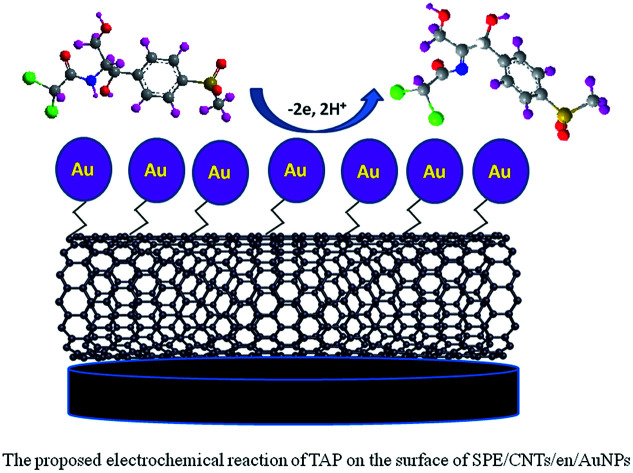

## 1. Introduction

Thiamphenicol [d(+)-*threo* 2,2*s*-dicholoroacetamido-1-(4-methylsulphonylphenyl) propane-1, 3-diol (TAP)] is an analogue of chloramphenicol in which the nitro group on the benzene ring is replaced with a methylsulphonic group.^1^ It is a broad-spectrum bacteriostatic antibiotic, active against both Gram positive and Gram negative pathogens, and also effective against anaerobic organisms.^2^ TAP has been used for therapeutic purposes on human and veterinary consumers. The prescription of TAP for animals has a major concern since the residues of TAP are found in dairy products for human consumption that thus enter the human body. Antibiotic residues have negative effects on consumers, adversely affecting the human body, leading to hypersensitivity, gastrointestinal erosions, ulcers, and kidney necrosis. Residues of antibiotics are said to have a toxicological effect during production of fermented products (*i.e.* cheese and yoghurt).^3^ To produce and distribute safe dairy products to consumers, a reliable, cost effective, and fast method needs to be developed for on-site monitoring of antibiotic residues.

Several methods, including capillary electrophoresis,^2^ liquid chromatography coupled with mass spectrometery,^4^ and high performance liquid chromatography, have been reported for the determination of TAP residues in various samples.^4^ These techniques have some advantages including high accuracy, precession, and robustness, but they also have some limitations for routine analysis in terms of cost, analysis time, sample size, and tedious sample pretreatment.^5^ Alternatively, electrochemical methods are characterized by simplicity, fastness, cost-effectiveness, and portability for quantitative analysis.^6^

The extraordinary electrochemical features of carbon nanotubes (CNTs) in terms of excellent electron transfer and large surface area^7^ make them suitable for use in modification of electrochemical sensors.^8,9^ Commonly, gold nanoparticles (AuNPs) are being used as a kind of nanomaterial for fabrication of nanocomposites and enhancement of sensitivity in electrochemical sensors because of excellent catalytic and conductivity properties.^10^

A nanocomposite of AuNP/CNTs is of particular interest owing to its easy fabrication process and wide potential application, moreover, it combines the excellent physicochemical properties of AuNPs and CNTs,^11^ which have been reported in several studies for electrochemical analysis.^12–14^ In general, AuNPs are decorated on the surface of CNTs by either a direct or indirect deposition process. In the direct deposition process, AuNPs are directly coated on the surface of CNTs by reduction of chloroauric acid. In the indirect deposition process, a covalent linkage is formed between AuNPs and CNTs in nanocomposite production.^15^ Efforts have been made to produce a AuNP/CNT nanocomposite *via* different techniques.^13,16,17^ A nanocomposite of AuNP/MWCNT was reported to facilitate electron exchange reactions with free-diffusing redox species.^18^ However, synthesis of this nanohybrid has been limited in some cases by the aggregation of AuNPs, which blocked the electro-active surface area of the CNTs, thereby reducing the catalytic effect of the resulting material.

In this study, we fabricated a nanocomposite of AuNPs and CNTs with ethylenediamine (en) as a linker between carboxyl groups of CNT and AuCl_4_^−^ for more distribution of gold ions prior to the fabrication process. The fabricated nanocomposite was used for modification of a screen printed electrode based on an electroless deposition process for voltammetric determination of thiamphenicol. To the best of our knowledge, this is the first report on the electrochemical determination of thiamphenicol in bovine milk samples using a nanocomposite (CNT/en/AuNP)-based electrochemical sensor.

## 2. Experimental

### 2.1. Apparatus and chemicals

All electrochemical measurements were carried out using a portable potentiostat (DropSens, μStat 8000, Spain) electrochemical system. The surface of the working electrode, *i.e.* the screen-printed carbon electrode (SPE, DropSens, Spain), was modified with CNT/en/AuNP and connected to the potentiostat using a USB connection. All characterization studies on the morphology and composition of the sensor were implemented by field emission scanning electron microscopy-energy dispersive spectroscopy (FESEM-EDS, JEOL, USA) and transmission electron microscopy (TEM, JEOL, USA). A pH meter (Fisher Scientific, USA) was used to set the pH values before each analysis.

Multi-walled carbon nanotubes (*D* × *L* 7–10 nm × 0.5–10 μm), ethylenediamine, and gold(iii) chloride hydrate (99.5%) were obtained from Aldrich (USA). Thiamphenicol (98%) was purchased from Tokyo chemical industry (TCI, Japan). All other chemicals were of analytical grade and used as received.

Milk samples were supplied from two different farms in Malaysia (Putra Mart UPM as farm A, and Johor as farm B) and kept in a fridge before analysis.

### 2.2. Pretreatment of the milk samples

The milk samples were treated using the method adopted by Moors and Massart with little modification.^19^ All milk samples were defatted and extracted in acetonitrile before analysis by the proposed electrochemical sensor. In the first step, all samples were spiked with TAP and mixed for 2 min on a magnetic stirrer. Then, acetonitrile was added drop wise under stirring to complete the deproteinization process. The cloudy sample was centrifuged for 10 min at 6000 rpm, and the supernatant was separated and evaporated by heating at 55 °C to decrease the volume to 1 mL. The final extracted sample was diluted to 2 mL with phosphate buffer and filtered through a filter paper (Whatman, 0.2 μm) before analysis.

### 2.3. Fabrication of the CNT/en/AuNP nanocomposite

**Scheme 1:**
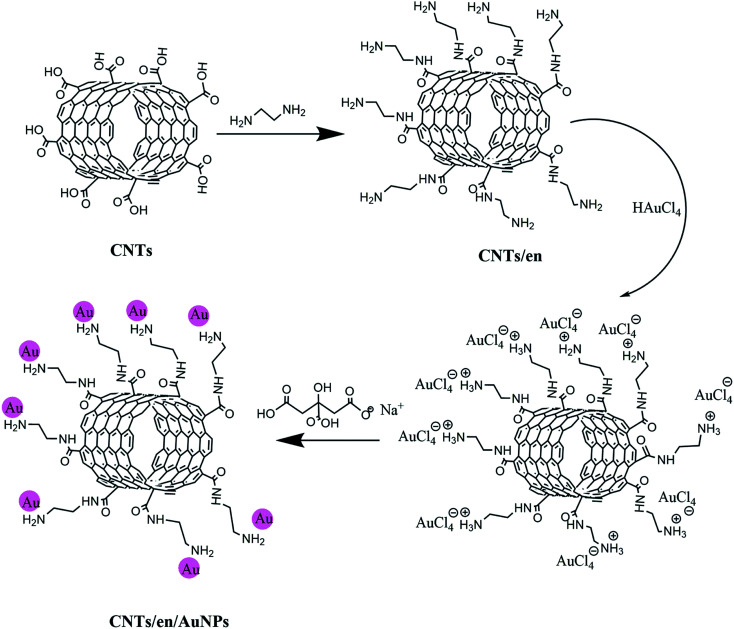
The chemical process for the synthesis of CNT/en/AuNP nanocomposite under reflux conditions.

The fabrication process for the synthesis of the nanocomposite consists of two steps. At first, 0.01 g of carbon nanotubes after carboxylation with nitric acid was suspended in 25 mL of an ethylenediamine/ethanol solution (0.1 M), and the reaction was completed under reflux conditions at 50 °C for 30 min. Then, HAuCl_4_ (0.1%, 1 mL) was added and refluxed for an additional 10 min. Sodium citrate (1% w/v, 2 mL) as a mild reducing agent was added drop wise to the reactor (see Scheme 1). The reduction of AuCl_4_^−^ to AuNPs was completed under reflux for 30 min, and the final solution was cooled and separated by centrifugation at 5000 rpm for 4 min. The sediment was dried in an oven at 55 °C for 2 h.

The proposed method for the fabrication of the electrochemical sensor was based on drop casting the synthesized nanocomposite on the surface of a working electrode (screen-printed electrode). For this purpose, 0.01 g of the synthesized nanocomposite (CNT/en/AuNP) was mixed with dimethylformamide (DMF, 10 mL) and dispersed in an ultrasonic bath for 30 min. Then, 5 μL of the suspension was drop casted carefully on the working electrode SPE and dried in air to evaporate the solvent.

## 3. Results and discussion

### 3.1. Morphological and elemental composition studies

**Fig. 1:**
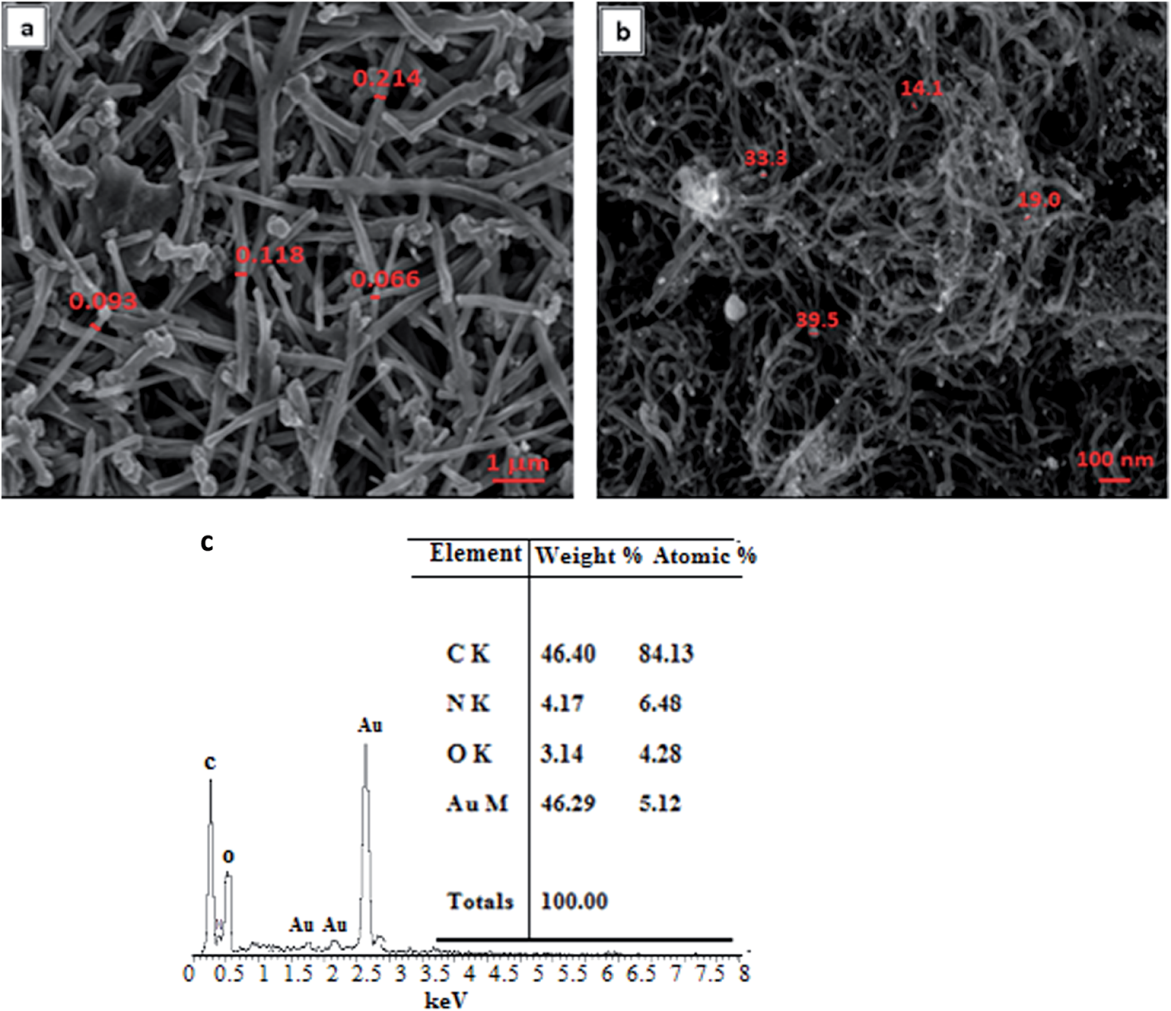
FESEM images of (a) CNTs and (b) CNT/en/AuNP and the respective EDS spectrum for the nanocomposite (c).

The surface morphology of the modified electrode was characterized by FESEM, as shown in Fig. 1a. A spaghetti-like structure of CNTs was observed on the SPE/CNT, whereas on the surface of SPE/CNT/en/AuNP (Fig. 1b), the distribution of bright spheres on the surface of carbon nanotubes appeared due to the dispersion of AuNPs on the surface of CNTs.

The composition of the modified electrode was studied using EDS. The result (Fig. 1c) shows the EDS result of CNTs after their decoration with AuNPs (CNT/en/AuNP) and their respective peaks for carbon (C), oxygen (O), nitrogen (N), and gold (Au) due to the covalent bonding of the amine group to the carboxylic group on the surface of CNTs and formation of AuNPs. The morphology of the synthesized nanocomposite was further evaluated by transmission electron microscopy. The result (Fig. S1-a†) shows the presence of carbon nanotubes on the surface of SPE, whereas Fig. S1-b† displays a high density and an even distribution of AuNPs on the surface of CNTs.

### 3.2. Electrochemical study of the SPE during the modification process

**Fig. 2:**
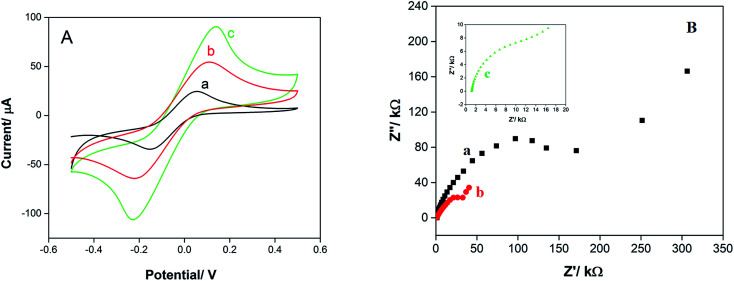
(A) CVs of bare SPE (a), SPE/CNTs (b), and SPE/CNT/en/AuNPs (c) in K_3_Fe(CN)_6_ (1 mM, 0.1 M KCl); (B) Nyquist plots for SPE (a), SPE/CNTs (b), and SPE/CNT/en/AuNPs (c) in a 1.0 mM K_3_Fe(CN)_6_ solution containing 0.1 KCl in the frequency range of 0.1 Hz to 100 kHz and amplitude of 0.01 V.

The electrochemical behavior of SPE in terms of surface area and conductivity during modification by CNTs and AuNPs was studied by cyclic voltammetry (CV) and electrochemical impedance spectroscopy (EIS), respectively. As shown in Fig. 2A-a, CV of K_3_Fe(CN)_6_ (1 mM, 0.1 M KCl) shows a pair of redox peaks due to the low electron transfer rate and limited surface area for SPE, whereas the peak currents increased after modification with CNTs (Fig. 2A-b) because of versatile properties CNTs to improve conductivity and surface area. The sensitivity enhancement of the electrodes increased further after modification with CNT/en/AuNP due to the synergistic effect between AuNPs and CNTs that caused an increase in electron transfer and effective surface area. The surface area of the prepared electrodes was calculated as 0.024 cm^2^, 0.033 cm^2^, and 0.050 cm^2^ for SPE, SPE/CNTs, and SPE/CNT/en/AuNPs, respectively, using the Randles–Sevcik equation.^20^

Electrochemical impedance spectroscopy (EIS) investigations of the electron transfer between an electrode surface and an electrolyte commonly contain a semicircular part (high frequency) and linear part (low frequency). The low frequency part of the plot shows the conductivity and electron transfer limited process. The semicircle diameter is equivalent to the charge transfer resistance (*R*_ct_), whereas the linear part at a higher frequency relates to the diffusion process of the electron transfer.^21,22^Fig. 2B shows the EIS results for the bare SPE (a), SPE/CNTs (b), and SPE/CNT/en/AuNPs using a solution containing a redox probe with 1.0 mM K_3_Fe(CN)_6_ and 0.1 M KCl. The results obtained from *R*_ct_ show that the modification of the electrode with CNTs (b) causes a significant decrease in *R*_ct_ in comparison with the case of bare SPE (a), from 127 kΩ for the bare to 5.45 kΩ for the SPE/CNTs; this indicates that pretreated MWCNTs has high conductivity and electron transfer. The *R*_ct_ of SPE/CNT/en/AuNPs (c) was obviously decreased to 0.75 kΩ as an evidence of the combination of AuNPs with carbon nanotubes.

### 3.3. Electrochemical behavior of TAP on the surface of a modified electrode

**Fig. 3:**
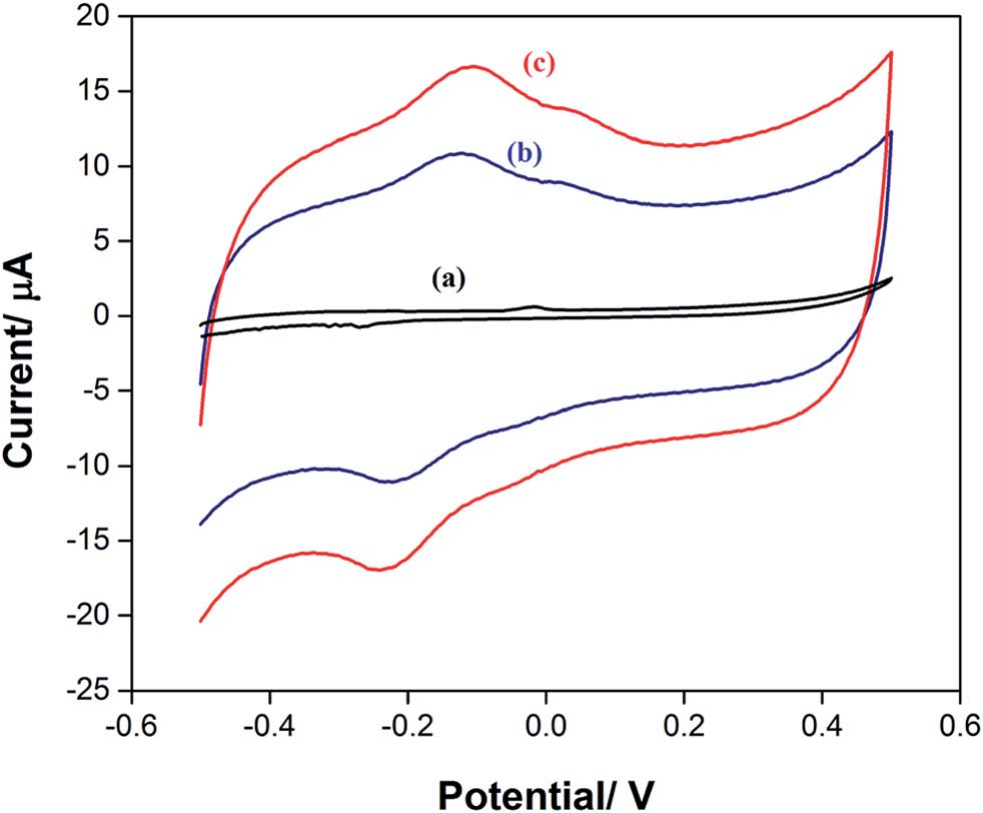
CVs of TAP (10 μM) on (a) SPE, (b) SPE/CNTs, and (c) SPE/CNT/en/AuNPs in 0.1 M citrate buffer (pH 6.0).

The cyclic voltammetry behavior of TAP in a 0.1 M citrate buffer solution (pH 6.0) on the surface of SPE, SPE/CNTs, and SPE/CNT/en/AuNPs was investigated. The result (Fig. 3a) shows a small irreversible redox peak for TAP on the bare SPE in the potential range from −0.5 to 0.5 V, revealing that TAP undergoes an irreversible redox process. The peak height increased after modification of SPE with CNTs (Fig. 3b) because of the excellent electron-transfer property and surface area that could be achieved by CNTs. As expected, the peak current of TAP was enhanced after modification with SPE/CNT/en/AuNPs (Fig. 3c). This further increase in peak height is attributed to the synergistic effect of AuNPs and CNTs as previously described.

**Fig. 4:**
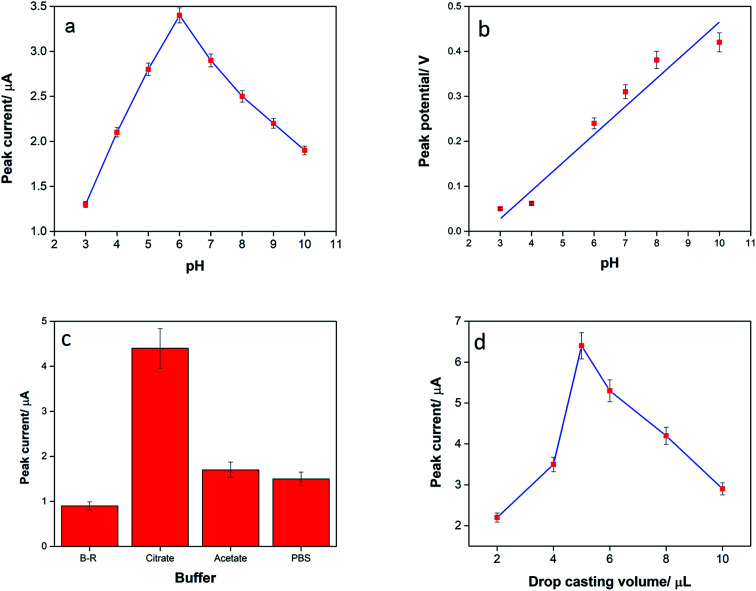
(a) The study of pH on the anodic peak current of 10 μM TAP in the range from 3 to 10, (b) relationship between peak potential and pH in the range from 3 to 10, (c) the effect of buffer on the oxidation peak current of TAP, and (d) the influence of the amount of CNT/en/AuNPs on the anodic peak current of TAP (10 μM). Each experiment was repeated three times.

The influence of pH on the oxidation peak of TAP was studied in 0.1 M citrate buffer in the range from 3 to 10 (Fig. 4a). It was observed that by increasing the pH from 3.0 to 6.0, the oxidation peak height increased, and then, the peak current declined. The results indicate that at pH > 6, the TAP molecule has a negative charge due to the de-protonation of amide group, and the repulsion forces with electrode surface decreases the mass transfer of TAP to the surface of electrode. It seems that by increasing the pH from 3 to 6, the adsorption process becomes predominant.^10,11^ As a result, pH 6.0 was chosen as the optimum pH for the electrochemical oxidation of TAP. The plot of *E*_p_ against pH (Fig. 4b) has a slope of 0.0590, close to the standard value of 0.060 V, revealing that the same number of H^+^ and e^−^ are participating in the oxidation of TAP.^11^

The kind of buffer on the anodic peak current of TAP was also studied at pH 6.0. The best result was obtained in citrate buffer (Fig. 4c); this showed that the TAP oxidation was more favored in a citrate buffer solution. Therefore, citrate buffer (0.1 M, pH 6.0) was chosen as the supporting electrolyte for practical analysis of TAP.

The relationship between the peak current of TAP and the amount of CNT/en/AuNPs on the SPE was also investigated. As a result, it was observed that by increasing the drop casting volume of nanocomposite suspension in the range of 2–10 μL, the oxidation signal of TAP increased due to the enhancement in surface area and complete covering of SPE. Inversely, the peak height decreased at >6 μL due to the thickness of the nanocomposite and decrease in conductivity (Fig. 4d).

**Fig. 5:**
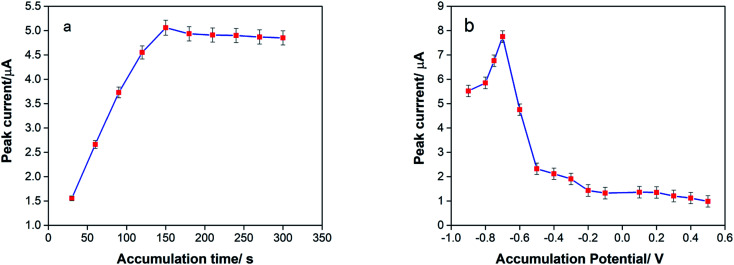
Effect of (a) accumulation time (b) accumulation potential on the oxidation peak current of 10 μM TAP on the surface of SPE/CNT/en/AuNPs in 0.1 M citrate buffer (pH 6.0). Each experiment was repeated three times.

In a further study, the influence of accumulation parameters (accumulation potential and accumulation time) was investigated on the sensitivity of fabricated electrochemical sensors to wards detection of TAP because of the effect of these parameters on the sensitivity of the electrochemical sensor during analysis.^23^ Accumulation time (*t*_acc_) was an effective parameter for the measured response of the proposed sensor. The effect of *t*_acc_ on the voltammetric detection of the analyte at the electrode was investigated between 30 and 300 s, as shown in Fig. 5a. The result shows that the anodic peak current enhanced after increasing the accumulation time of TAP on the surface of SPE/CNT/en/AuNPs in the range from 30 to 150 s. The peak current leveled off at *t*_acc_ > 150 s due to the saturation of TAP on the electrode surface. Therefore, *t*_acc_ of 150 s was selected as the optimum preconcentration time for TAP analysis.

During the preconcentration step on the oxidation of TAP, it was shown that the accumulation potential (*E*_acc_) was an effective parameter to quantify the response of the sensor. The effects of *E*_acc_ on the voltammetric determination of TAP at the AuNP/en-MWCNTs electrode were evaluated in the potential range from 0.2 to −0.9 V. CV was performed between −0.5 and 0.5 V at a scan rate of 50 mV s^−1^, measurements were conducted, and results were obtained. The result (Fig. 5b) shows that by applying the *E*_acc_ from 0.2 to −0.7 V, the peak current of TAP increases sharply due to the fact that TAP molecules adsorb better on a more negatively charged surface. Moreover, the reduced state of TAP accumulated on the electrode surface, and its oxidation peak current is bigger. However, at *E*_acc_ < −0.7 V, the peak current declined due to the instability of the electrode surface during hydrogen evolution. Therefore, *E*_acc_ of −0.7 V was chosen as the optimum value for the sensor to reach higher sensitivity during electro-oxidation of TAP.

### 3.4. Study on the electrochemical reaction of TAP

**Fig. 6:**
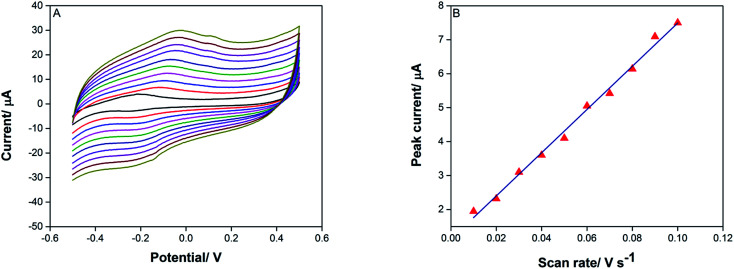
(A) Cyclic voltammograms for the oxidation of 10 μM TAP on the surface of SPE/CNT/en/AuNPs in 0.1 M citrate buffer (pH 6.0), accumulation potential −0.7 V, and accumulation time 150 s. (B) Linear plot of 10 μM TAP *versus* oxidation peak current at SPE/CNT/en/AuNPs.

To realize the electrochemical reaction of TAP in terms of reversibility, the number of electron transfers, and Tafel plots, cyclic voltammetry of TAP was conducted at *n* the scan rates of 0.010–0.1 V s^−1^. The result (Fig. 6A) shows that the anodic peak current of TAP increases linearly with the increasing scan rate with a linear equation of *I*_pa_ (μA) = 63.645*ν* (V s^−1^) − 1.126 (*r* = 0.9943) (Fig. 6B), suggesting that the electrode process is under adsorption control.

Furthermore, there was a good linear relationship between log *I*_pa_ and log *ν* with a corresponding equation that is expressed as log *I*_pa_ (μA) = 0.941 log *ν* (V s^−1^) + 0.935; (*R*^2^ = 0.991). The slope of 0.941 is close to the expected theoretical value of 1.0 that is typical of an adsorption control process.^24^

**Scheme 2:**
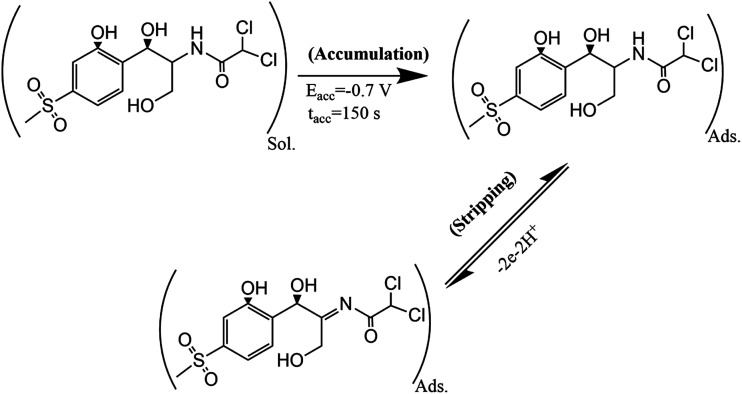
The proposed mechanism for determination of TAP based on the adsorptive stripping voltammetry on the surface SPE/CNTs/en/AuNPs.

In eqn (1), *I*_pa_ is anodic peak current (μA), *n* is electron transfers number, *F* is a constant value (96 450 C mol^−1^), *Q* (C) is the charge of electrode during TAP oxidation, and *ν* (V s^−1^) is the scan rate of cyclic voltammogram. The value of *n* has been calculated from the slope of *I*_pa_*vs. ν* as 1.86 ≈ 2.0; this means that two electrons are attributed to the oxidation of TAP. Hence, it can be concluded that the oxidation reaction of TAP at SPE/CNT/en/AuNPs involves two protons and two electrons (Scheme 2). Moreover, Fig. 6A shows that the oxidation peak potential (*E*_p,a_) shifted to anodic values with the increasing scan rate. As a theoretical concept in irreversible electrochemical reactions, the plot of *E*_p_*versus* log *ν*, where *ν* is scan rate, yields a straight line with the slope of 
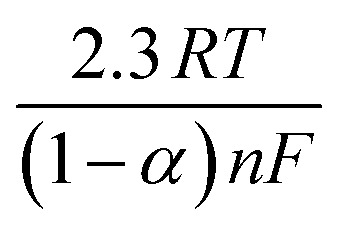
 for the anodic currents.^25,26^ As a result, the value of *α* (electron transfer coefficient) was estimated to be 0.31, showing asymmetry of the transition state energy of TAP during oxidation on the surface of the modified electrode.1
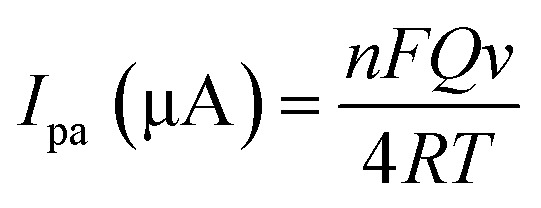


**Fig. 7:**
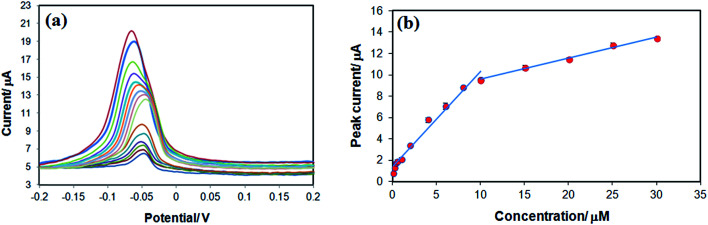
(a) Differential pulse voltammetry of TAP at different concentrations. (b) Calibration curve of TAP at −0.05 V in 0.1 M citrate buffer (pH 6.0); accumulation time, 150 s; accumulation potential, −0.7 V, and scan rate, 0.1 V s^−1^.

In a further study, differential pulse voltammetry (DPV) was employed to plot the calibration curve of the fabricated sensor against TAP concentration under the optimum conditions. The results (Fig. 7a) showed that the oxidation peak potential shifted around 10 mV to negative direction due to the faster of electron transfer with the surface of the electrode in the presence of adsorbed TAP. As shown in Fig. 7b, there are two linear calibration ranges between anodic peak current and TAP concentration (0.1–10 μM and 10–30 μM) with the equations of *I*_pa_ (μA) = 0.9888*C* + 1.2563 (*R* = 0.9948) and *I*_pa_ (μA) = 0.216*C* + 7.36 (*r* = 0.9972), respectively (Fig. 7b). This observation is due to the saturation of some activation sites on the surface of modified electrode at concentrations more than 10 μM.^27^ The limit of detection (LOD) and limit of quantification (LOQ) were 0.003 μM and 0.01 μM, respectively, based on the following equations:LOD = 3*Sm*^−1^ and LOQ = 10*Sm*^−1^; (*S*, standard deviation of blank solution; *m*, slope of calibration curve).

### 3.5. Reproducibility, repeatability, and stability studies

The repeatability of the proposed sensor in the determination of TAP was evaluated by performing five successive determinations with the same solution of TAP (10 μM) using the same fabricated sensor. The results have a relative standard deviation (RSD) of 3.6% for the response of the electrode towards 10 μM TAP. Reproducibility of the electrode was also studied by preparing six different electrodes by the same procedure and using them for the determination of TAP. The results with the RSD of 2.1% showed good reproducibility for the fabrication process of the TAP sensor.

The stability of the fabricated sensor was evaluated on a daily basis for a period of twelve days. The results showed that the electrode retained 90% of its initial signal for the determination of 10 μM TAP within seven days, and then, the response decreased by 15% up to 12 days. These studies indicate that the fabricated sensor is repeatable and reproducible with satisfactory stability and can be applied as a reliable sensor for the analysis of TAP content in milk samples.

### 3.6. Study on the selectivity of the fabricated sensor

The effect of some possible co-existing compounds in bovine milk in the presence of TAP was studied on the electrochemical signal of TAP under optimized experimental conditions. As shown in Table 1 and Fig. S2 (ESI data†), 50 times excess of amoxicillin, penicillin G, ampicillin, 15 times excess of florfenicol, 100 times excess of lactose, casein protein, and 200 times excess of some cations (K^+^, Ca^2+^, Mn(ii), Mg(ii), Ca(ii), Fe(ii), and Zn(ii)) have no significant interference on the signal of TAP. Moreover, an excess of a quadratic mixture of amoxicillin, penicillin G, ampicillin, and +Florfenicol changed 5.0% of the thiamphenicol signal. This suggests that the fabricated sensor has a satisfactory selectivity for determination of TAP in milk samples.

**Table 1:** The effect of some co-existing compounds on the determination of TAP (10 μM)

Compounds	Tolerance^a^ (mol/mol)	Current change (%)
Amoxicillin	50	+4.8
Penicillin G	50	+4.6
Ampicillin	50	+4.7
Sulfadiazine	100	+2.8
Florfenicol	15	+8.2
(Amoxicillin, penicillin G, ampicillin, florfenicol, sulfadiazine)	40 : 45 : 50 : 12 : 85	+5.0
Lactose	100	+3.4
Casein protein	100	+3.6
K^+^	200	+4.5
Ca^2+^	200	−4.0
Mg(ii)	200	−4.6
Zn(ii)	200	−4.8
Mn(ii)	200	−4.7
Fe(ii)	200	−4.0

a The maximum mol ratio of each species that cause ≤5% change in the determination of TAP.

### 3.7. Application of the proposed sensor

The accuracy of the fabricated sensor (SPE/CNT/en/AuNPs) was studied by determination of TAP in spiked fresh milk samples obtained from dairy farms in Malaysia. All obtained samples were pretreated based on the method described in Section 2.2. Table 2 shows that all recoveries are in the range from 94% to 97% due to high selectivity of the proposed TAP sensor and adequate pretreatment of milk samples before analysis.

**Table 2:** The results of TAP analysis in some bovine milk samples obtained using the proposed sensor

Sample	Spiked (μM)	Found (μM)	Recovery (%)
Fresh milk^a^	—	<LOD	—
	1.0	0.943 ± 0.020	94.3
	2.0	1.930 ± 0.010	96.5
Fresh milk^b^	—	<LOD	—
	1.0	0.957 ± 0.010	96.0
	2.0	1.94 ± 0.010	97.0

a Farm A.

b Farm B.

## 4. Conclusion

In this study, we combined the advantages of good conductivity, small size, and large surface area of CNTs and the catalytic property of AuNPs to fabricate an electrochemical sensor sensitive to thiamphenicol residue in milk samples. In the fabrication process, ethylenediamine covalently bonded to carboxylate groups on CNTs and the other amine group electrostatically bonded to AuCl_4_^−^ in acidic media. The process was completed after reduction of AuCl_4_^−^ in the presence of sodium citrate. The modified electrode (SPE/CNT/en/AuNPs) showed excellent electrocatalytic activity and remarkable sensitivity towards the oxidation peak current of TAP. The fabricated sensor displayed good operating characteristics including reproducibility, repeatability, stability, a low detection limit, and a wide linear dynamic range for the detection of TAP. Finally, reliability and accuracy of the proposed sensor were studied in milk samples spiked with TAP. The output results in the analysis of spiked TAP samples in fresh milk show the suitability of the proposed sensor for the detection of TAP residues.

## Conflicts of interest

There are no conflicts to declare.

## Supplementary Material

RA-008-C7RA07544H-s001
