# Supplementary material for: A screen printed carbon electrode modified with carbon nanotubes and gold nanoparticles as a sensitive electrochemical sensor for determination of thiamphenicol residue in milk
Source: RSC Adv. 2018 Jan 11;8(5):2714–22. doi: 10.1039/c7ra07544h (PMC9077468; doi:10.1039/c7ra07544h)
Supplement: RA-008-C7RA07544H-s001 [file RA-008-C7RA07544H-s001.pdf]

Electronic Supplementary Material (ESI) for RSC Advances.
This journal is © The Royal Society of Chemistry 2017

Screen Printed Carbon Electrode Modified with Carbon Nanotubes and Gold Nanoparticles as a Sensitive Electrochemical Sensor for Determination of Thiamphenicol Residue in Milk

Supplementary data

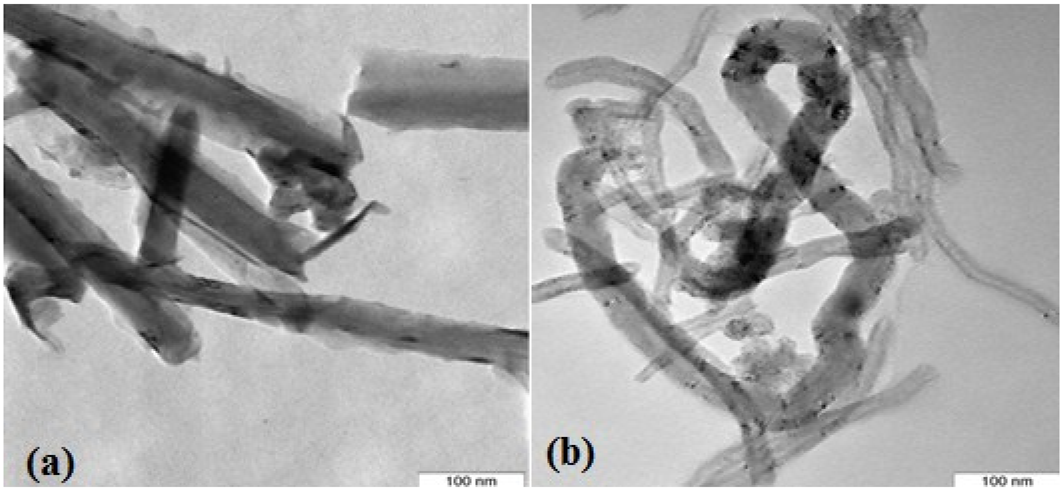

Fig. S1: TEM images of (a) SPE/CNTs and (b) SPE/CNTs/en/AuNPs.

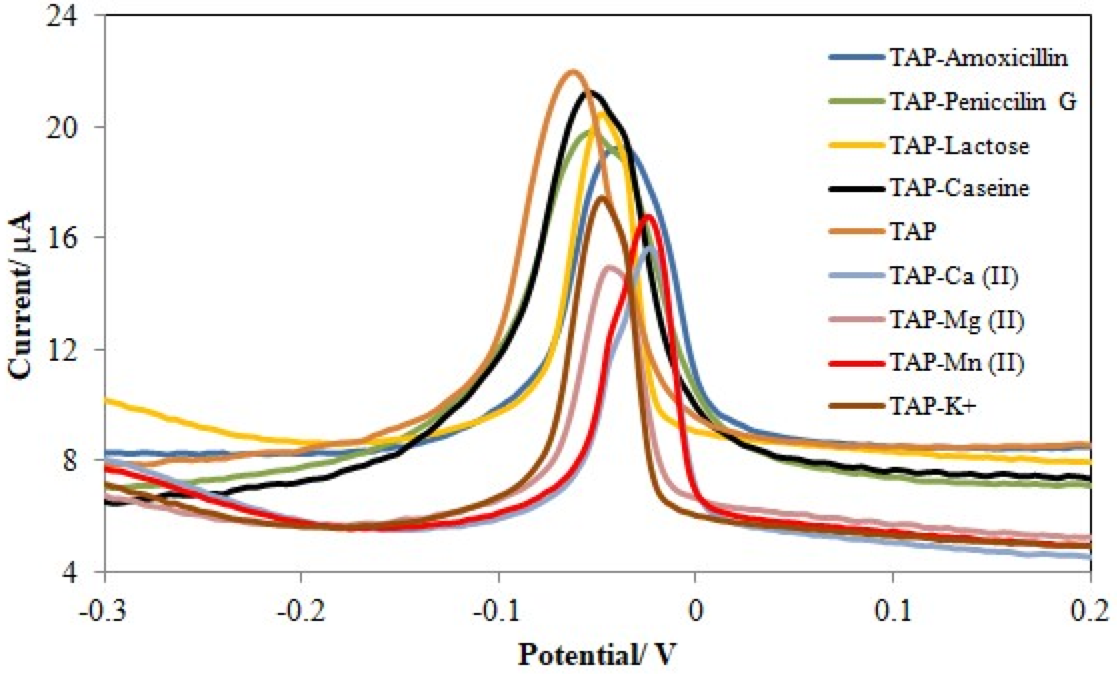

Fig. S2: Voltammograms of thiamphenicol in the presence of different co-existing compounds and cations at 2 mM. Thiamphenicol: 10 µM.
